# Development of EST-Molecular Markers from RNA Sequencing for Genetic Management and Identification of Growth Traits in Potato Grouper (*Epinephelus tukula*)

**DOI:** 10.3390/biology10010036

**Published:** 2021-01-07

**Authors:** Te-Hua Hsu, Yu-Ting Chiu, Hung-Tai Lee, Hong-Yi Gong, Chang-Wen Huang

**Affiliations:** 1 Department of Aquaculture, National Taiwan Ocean University, Keelung 20224, Taiwan; realgigi@mail.ntou.edu.tw (T.-H.H.); andychiou1@gmail.com (Y.-T.C.); hygong@mail.ntou.edu.tw (H.-Y.G.); 2 Center of Excellence for the Oceans, National Taiwan Ocean University, Keelung 20224, Taiwan; 3 Department of Environmental Biology and Fisheries Science, National Taiwan Ocean University, Keelung 20224, Taiwan; hungtailee@gmail.com

**Keywords:** broodstock, full-sib family, transcriptome, SNPs, microsatellites

## Abstract

**Simple Summary:**

The potato grouper is a novel aquaculture species in Taiwan. Due to the lack of genetic information concerning this species, we have developed molecular markers based on transcriptome sequencing and further characterized their association with gene diversity and growth traits of this species. Ultimately, these markers could be utilized as accurate and efficient tools for genetic management and marker-assisted selection of potato grouper with distinct growth traits.

**Abstract:**

The accuracy and efficiency of marker-assisted selection (MAS) has been proven for economically critical aquaculture species. The potato grouper (*Epinephelus tukula*), a novel cultured grouper species in Taiwan, shows large potential in aquaculture because of its fast growth rate among other groupers. Because of the lack of genetic information for the potato grouper, the first transcriptome and expressed sequence tag (EST)-derived simple sequence repeat (SSR) and single nucleotide polymorphism (SNP) markers were developed. Initially, the transcriptome was obtained from seven cDNA libraries by using the Illumina platform. *De novo* transcriptome of the potato grouper yielded 51.34 Gb and 111,490 unigenes. The EST-derived SSR and SNP markers were applied in genetic management, in parentage analysis, and to discover the functional markers of economic traits. The F_1_ juveniles were identified as siblings from one pair of parents (80 broodstocks). Fast- and slow-growth individuals were analyzed using functional molecular markers and through their association with growth performance. The results revealed that two SNPs were correlated with growth traits. The transcriptome database obtained in this study and its derived SSR and SNP markers may be applied not only for MAS but also to maintain functional gene diversity in the novel cultured grouper.

## 1. Introduction

With the increase in the global population, the annual demand for animal-based protein has also risen, which includes increasing requirements for aquaculture. Aquaculture is critical for providing superior animal protein to humans [1]; thus, efficient aquaculture production is essential. While domesticated animals have long breeding histories, many farmed aquatic species (especially marine fish) are still in the early stages of domestication; many seeds are still obtained from the wild [2]. Thus, novel aquaculture species based on phenotype and genotype selection can improve individual phenotypes, yielding traits that enhance economic performance without additional production cost [3].

The accuracy and efficiency of marker-assisted selection (MAS) has been proven in the selection of economically essential aquaculture species [4]. Complete broodstock management and breeding programs assist the hatchery by decreasing the cost, workforce, and feed [5,6]. Conventional long-term selective breeding programs, such as those of Atlantic salmon (*Salmo salar*) [7], rainbow trout (*Oncorhynchus mykiss*) [8], and Nile tilapia (*Oreochromis niloticus*) [9], may cause stock inbreeding because candidate broodstock is always chosen from a few phenotypes of interest (e.g., fast growth) [10]. This impairs genetic diversity and may cause an unexpected loss of essential characteristics, such as disease resistance [11,12]. Inbreeding and low genetic diversity can be minimized by the systematic selection of strains or families with a breeding record [13,14,15]. The genetic breeding programs of Atlantic salmon in Norway are an outstanding example of economic aquaculture. Large-scale commercial production systems have been established based on genetic management. The use of MAS in Atlantic salmon breeding programs increases efficiency [16,17,18] and has allowed various vital genetic traits to be improved and combined [19,20].

Groupers consist of several genera in the subfamily Epinephelinae (family: Serranidae). Economically, they are popular aquaculture species, with approximately 4.7 million tons of groupers being cultured and captured every year [21]. East and Southeast Asian countries are major production areas of groupers, and approximately 92% of the world’s grouper production was in Asia [22]. The potato grouper (*Epinephelus tukula*) is a novel and economic cultured grouper species, and its aquaculture is still in the early development stage. The International Union for Conservation of Nature and Natural Resources (IUCN) has listed the potato grouper as a nonendangered species due to its indefinite economic value and lack of biological information [23]. The advantages of fast growth in juvenile and adult potato groupers are expected to yield benefits [24]. Because of their large bodies, the broodstocks, which mainly comprise first-generation wild-type fish, are usually reared without tags in outdoor cement ponds. Moreover, combining their dominant traits through hybridization of different species (e.g., *E. fuscoguttatus* × *E. lanceolatus*) is a common practice in the Asian grouper industry [25]. Therefore, the potato grouper, or one of the new hybrid species, may be the next widely cultured species in the aquaculture industry. Even though grouper is a hermaphroditic species with a long generation interval, it is possible to not only plan a long-term breeding program by artificial selection but also carry out commercial production [26,27,28].

Genetic information is still scarce for the potato grouper. Therefore, in this study, we developed the first transcriptome and expressed sequence tag (EST)-derived SSR and single nucleotide polymorphism (SNP) markers. These markers were used in genetic management, in parentage analysis, and to discover functional markers with economic traits. EST markers enable the continuation of diverse functional genes during genetic selection. Furthermore, the findings of the present study will allow fast and cost-effective breeding of numerous full-sibs and half-sibs as well as novel strain grouper species [29,30].

## 2. Materials and Methods

### 2.1. Samples Preparation

**Figure 1:**
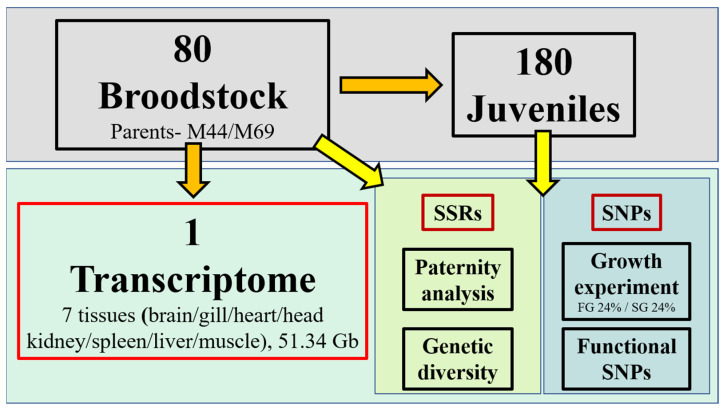
Experimental diagram showing the identification of molecular markers ideal for genetic management (parentage analysis and genetic diversity) and marker-assisted selection (MAS) of potato groupers. 80 broodstock individuals of potato grouper were tagged and cultured. A mature potato grouper was randomly chosen for transcriptome analysis. Seven tissues (the brain, gill, heart, head kidney, spleen, liver, and muscle) were collected for RNA sequencing. 180 juveniles were used for growth experiments and divided into two groups: the fast-growth group (top 24%; FG) and the slow-growth group (bottom 24%; SG). Simple sequence repeats (SSRs) were used for paternity analysis and genetic diversity; single nucleotide polymorphisms (SNPs) were used for the growth experiment and functional SNPs. Parents- M44/M69 represents the tagged id of broodstock.

In this study, potato groupers were collected from the grouper stock hatchery (Long Diann Marine Biotechnology, Pingtung, Taiwan). A total of 80 broodstock individuals of potato grouper were tagged and cultured in a 500-ton outdoor cement pond since 2012. A mature potato grouper (body weight (BW), 14 kg; body length (BL), 91 cm; total length (TL), 100 cm) was randomly chosen and anesthetized for transcriptome analysis. Seven tissues (the brain, gill, heart, head kidney, spleen, liver, and muscle) were collected and fully immersed in RNA TriPure isolation reagent (Roche Diagnostics, Indianapolis, IN, USA) and maintained at −80 °C for RNA sequencing (Figure 1).

In 2018, the first fertilized eggs were collected and cultured in the hatchery. After 2 months of rearing, 200 juveniles (TL 3.3 cm) were randomly collected and transported from the hatchery to National Taiwan Ocean University (NTOU). Then, 200 juveniles were cultured in 60 × 45 × 45 cm^3^ (length × width × depth) glass fish tanks with a recirculation system. The water temperature was kept at 26–28 °C, the salinity was maintained at 32–35‰, and the fish were fed with commercial feed three times per day (9:00 am, 3:00 pm, and 9:00 pm). Next, 180 of 200 individuals were randomly chosen and allocated to six tanks for growth experiments. After a week of acclimatizing, growth-related traits, including BW, BL, and TL, were measured for 180 juveniles for 90 days of culture. The brain, liver, and muscle tissues of the three largest and three smallest fish were collected after 90 days and immersed separately in RNA TriPure isolation reagent (Roche Diagnostics, Indianapolis, IN, USA) and maintained at −80 °C. Caudal fins were collected from each individual and preserved in 70% ethyl alcohol at −20 °C. All juveniles were divided into two groups: the fast-growth group (top 24%; FG) and the slow-growth group (bottom 24%; SG) (Figure 1).

One 5 mm and three 3 mm stainless steel beads were added to a microtube with Trizol and tissues, and the mixture was homogenized in a SpeedMill PLUS high-speed tissue homogenizer (Analytik Jena AG, Jena, Germany). Total RNA of each tissue was extracted using an EasyPure Total RNA spin kit (Bioman, Taipei, Taiwan), and genomic DNA (gDNA) was extracted using a Gene-SpinTM Genomic DNA isolation kit (Protech Technology Enterprise, Taipei, Taiwan) following manufacturer’s instructions. The quality and quantity of total RNA and gDNA were determined using Nanodrop One (Thermo Fisher Scientific, San Jose, CA, USA) and run on 0.8% agarose gel. All samples of gDNA were diluted to 25 ng/µL for the DNA template and maintained at −20 °C. All tissues of total RNA were reverse-transcribed to cDNA by using a high-capacity cDNA reverse transcription kit (Applied Biosystems, Foster City, CA, USA) and maintained at −80 °C.

### 2.2. De Novo Assembly, Annotation, and Marker Detection

Total RNA (2 µg) from the seven tissues (brain, gill, heart, head kidney, spleen, liver, and muscle) were separately sequenced, and seven cDNA libraries were established to construct a transcriptome of the potato grouper, followed by RNA-seq using the Illumina sequencing platform [31]. Agilent 2100 Bioanalyzer (Agilent Technologies, Santa Clara, CA, USA) and ABI StepOnePlus real-time PCR system (Applied Biosystems) were used to qualify and quantify the cDNA libraries. Finally, these cDNA libraries were sequenced using the Illumina HiSeq 4000 platform.

From the raw reads, the following were filtered out: adaptors, reads with >5% unknown nucleotides, and low-quality sequences. The transcriptome assemblies of clean reads from the seven tissues were separately acquired using Trinity software [32]. To obtain the integrated unigene, Tgicl v2.1 software was used to cluster and assembly the unigenes into a large EST database [33]. *De novo* transcriptome assembly of seven libraries was submitted to the NCBI short read archive database (accession numbers: SRR12853319, SRR12854351, and SRR12855035-39).

The numbers of functional unigene sequences in deference libraries were subjected to BLAST search and annotated against the NCBI nucleotide sequences (Nt), nonredundant (Nr) protein of four related and some other fish species, gene ontology (GO) [34], clusters of orthologous groups (COG), Kyoto Encyclopedia of Genes and Genomes (KEGG), and SwissProt databases. Subsequently, the classification of all unigenes into GO, COG, and KEGG categories was annotated with the Blast2GO [35] and BLASTx software with an E-value threshold of 10^−5^. Unigenes were compared with the nucleic acid database nucleotide through BLASTn (NT) (*p* < 0.00001): the protein with the highest sequence similarity to the unigene and the function annotation information of the unigene protein were both obtained.

All types of microsatellites, from mononucleotides to hexanucleotides, were detected using MISA version 1.0.0 software [36]. The parameters were determined to identify mono-, di-, tri-, tetra-, penta-, and hexanucleotides with a minimum of 12, 6, 5, 5, 4, and 4 motifs, respectively. The sequences of each microsatellite were used to design five sets of primers using Primer3 software [37].

SNPs were detected in each library using SAMtools and Picard software, which compare data with sequencing genomic locus and repeated reads. Next, HISAT software [38] was used to align the clean reads to unigenes. GATK v4.0 software [39] was used to improve the SNPs and InDel calling and eliminate low-quality SNPs.

### 2.3. SSR Analysis

The target PCR product was amplified in two steps, as recommended by Schuelke [40]. All forward primers and four fluorescent dyes (FAM, JOE, NED, and ROX) were labeled with an adaptor sequence. A total of 14 nonfunctional SSR [41,42,43] and 67 functional SSR primers (Table S7) were redesigned by using a labeling adaptor. For the first step, 10 µL of the PCR reaction mixture containing 5 µL of *Taq* DNA polymerase 2Χ Master Mix RED (Ampliqon, Odense M, Denmark), 0.3 µL of forward primer (10 µM), 0.3 µL of reverse primer (10 µM), 2 µL of template DNA (25 ng/µL), and 2.4 µL ddH_2_O. PCR was performed at 95 °C for 5 min, followed by 30 cycles of 95 °C for 40 s, T_A_ °C for 30 s, and 72 °C for 40 s, ending with 72 °C for 5 min. For the second step, the allocated mixture was the same as above, except that the forward primer and template DNA were exchanged for the fluorescent primer (10 µM) and the first PCR product (diluted 10 times), respectively. The PCR procedure was also performed under the same conditions as the first step. The amplicons ranged from 110 to 410 bp, and PCR products (2 µL) were checked for by running on 2% agarose gel and dyeing in GelRed^®^ nucleic acid gel stain (Biotium, Hayward, CA, USA) for 30 min.

Multiple PCR products of each identical sample containing different fluorescent dyes were pooled into a 96-well microplate. The SSR fragments were separated using an ABI PRISM^®^ 3730xl DNA analyzer instrument (Applied Biosystems) with capillary electrophoresis. The output data were analyzed using GeneMapper^®^ v4.0 software (Applied Biosystems).

### 2.4. MassARRAY

In total, 46 SNPs were obtained from the transcriptome database genotyped by Agena MassARRAY platform and iPLEX chemistry (Agena, San Diego, CA, USA). All specific and extension primers (Table S7) were designed using Assay Designer v.4.0. PCR amplification and genotyping were performed using Ellis and Ong’s method [44]. Briefly, 7 nL of purified primer extension reaction was loaded onto a matrix pad of a SpectroCHIP (Agena). SpectroCHIPs were analyzed using MassARRAY Analyzer 4, and calling was performed through clustering analysis with TYPER 4.0 software. The genotype call rate was used to determine the accuracy of the result based on the following formula: P_MA_ × P_YLD_ × P_SKW_, where P_MA_-peak represents the correction of molecular weight and signal sharpness, P_YLD_-peak represents signal strength, and P_SKW_-SNP represents the signal strength ratio between two SNP genotypes. According to the call rate ranging from low to high, the genotype was assigned “aggressive,” “moderate,” or “conservative,” in that order. AutoCluster (https://www.geneticaffairs.com/features-autocluster.html) was used to cluster homozygotes and heterozygotes into two groups and plot a two-dimensional graph with the peak signal as the coordinate axis. The SNP genotypes were recognized through clustering.

### 2.5. Statistical Analysis

Geneious software (https://www.geneious.com/) was used to analyze the genotypes of markers with multiple fluorescent polymorphic amplicons. The genotypes of each SSR marker were imported into the GenAlEx software [45], which is fully compatible with Excel for Windows. For statistical analysis of population diversity, the parameters related to the number of alleles (N_A_) and allele frequency (N_E_) were as follows: observed heterozygosity (*H_O_*), expected heterozygosity (*H_E_*), polymorphism information content (PIC), fixation index (*F_IS_*), and Hardy–Weinberg equilibrium (HWE) [46,47,48].

SPSS v22.0.0 (ICM) was used for one-way analysis of variance to determine the significance of correlations between genotypes of molecular markers (SSR and SNPs) and strains. Parentage analysis was analyzed using PARFEX v1.0 [49].

### 2.6. Quantitative Real-Time PCR

The cDNA of each individual was diluted 50 times as a template for quantitative real-time PCR (qRT–PCR). The specific primers (Table S7) were designed using Primer3web (http://bioinfo.ut.ee/primer3/). qRT–PCR was performed using Power SYBR^®^ Green PCR Master Mix (Thermo Fisher Scientific) on a Roche LightCycler^®^ 480 Instrument II (Roche Applied Science). All experiments were performed in triplicate. The expression levels of each gene were normalized to the expression of an internal housekeeping control, namely beta-actin. The median in each triplicate was used to calculate the relative target gene concentrations (Cp = Cp median target gene-Ct median beta-actin). The relative quantification was calculated and performed following Schmittgen and Livak’s method [50].

## 3. Results

### 3.1. RNA-Seq

By using the Illumina HiSeq 4000 platform, 342.27 M high-quality clean reads with a length of 150 bp were yielded from the seven cDNA libraries after data filtering, including 48.33 M in the brain, 49.10 M in gill, 49.46 M in the head kidney, 48.68 M in the heart, 48.14 M in the liver, 49.13 M in the spleen, and 49.45 M in the muscle (Table S1). After the elimination of redundancies, 51.34 Gb was generated with an average of 7.33 Gb in each library, including 7.25, 7.36, 7.42, 7.30, 7.22, 7.37, and 7.42 Gb, respectively. A total of 111,490 unigenes were assembled in seven libraries, with an average total length of 1549 bp (seven tissues; N50: 3358 bp), including 1402 bp (brain; N50: 2898 bp), 1162 bp (gill; N50: 2242 bp), 1199 bp (head kidney; N50: 2288 bp), 1187 bp (heart; N50: 2322 bp), 876 bp (liver; N50: 1601 bp), 1238 bp (spleen; N50: 2478 bp), and 753 bp (muscle; N50: 1253 bp), respectively (Table S2). The size distribution indicated that the length of the 48,427 unigenes was >1000 bp (Figure S1).

In this experiment, 111,490 unigenes were assembled based on the pooled transcripts from the seven tissues of the potato grouper. To annotate these unigenes, we used BLASTx against the Nr, Nt, SwissProt, COG, KEGG, and GO databases, which were successfully annotated for 56,638 (50.80%), 65,703 (58.93%), 52,871 (47.42%), 23,718 (21.27%), 51,262 (45.98%), and 24,368 (21.86%) unigenes, respectively (Table S3). After cross-comparison and analysis of Venn diagrams, the number (proportion) of genes annotated to any database was 72,634 (65.15%), and the number of genes that could be simultaneously annotated in the six databases was 10,944 (9.82%); in addition, the number (proportion) of genes annotated in the five major databases other than GO or COG were 10,603 (9.51%) and 10,160 (9.11%), respectively. The results revealed that the number of genes annotated to the database from the seven tissues of the potato grouper was approximately 21,104–21,547.

Functional annotation information of the assemblies included unigene protein and COG functional categories. We aligned the unigene sequence through BLASTx and then aligned the annotated genes in the nonredundant protein database (Nr) to large yellow croaker (*Larimichthys crocea*) (36.08%), damselfish (*Stegastes partitus*) (20.56%), leatherhead Antarctic fish (*Notothenia coriiceps*) (7.85%), Nile tilapia (*Oreochromis niloticus)* (6.01%), and other species (29.49%) (Figure S2).

#### 3.1.1. Functional Annotation

**Figure 2:**
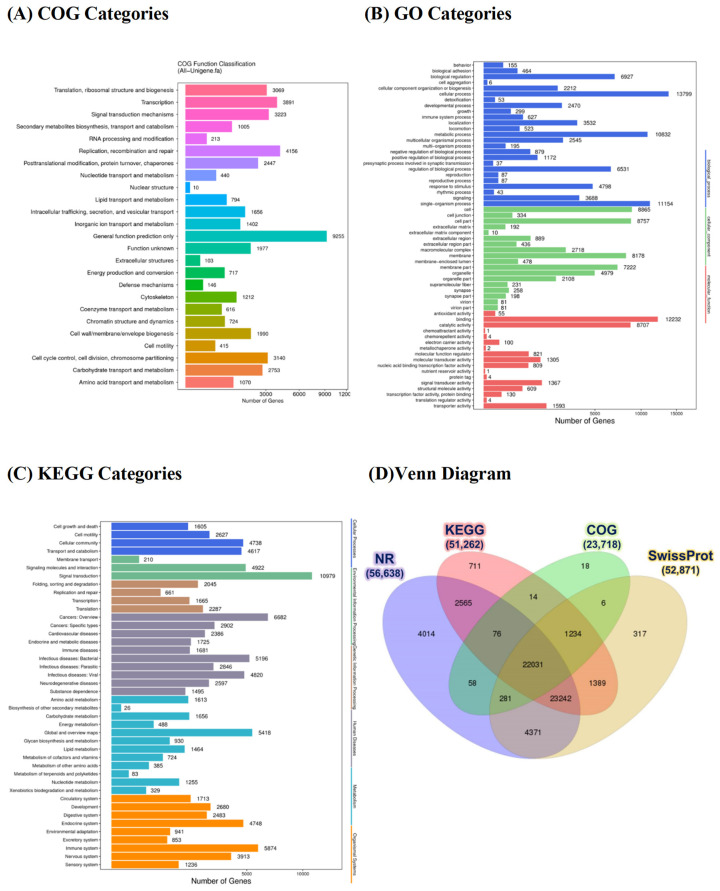
Histogram of clusters of orthologous groups (COG) (**A**), gene ontology (GO) (**B**), and eukaryotic cluster ortholog groups classification (KEGG) (**C**) functional annotations. Venn diagram (**D**) showing common and specific genes from the nonredundant (NR), KEGG, COG, and SwissProt databases.

The assembled unigene sequences of the potato grouper were subjected to BLAST searching against COG, GO, and KEGG databases. Figure 2A–C summarizes the statistical results. The possible functions of unigenes were predicted and classified by searching their predicted coding sequences (CDSs) of unigenes against the COG database. Possible functions of 23,718 unigenes were classified and subdivided into 25 COG categories (Figure 2A; Table S4), among which the cluster “General function prediction only” was the largest group (9255 unigenes), followed by “Replication, recombination and repair” (4156 unigenes) and “Transcription” (3891 unigenes). The three smallest clusters were “defense mechanisms” (146 unigenes), “extracellular structures” (103 unigenes), and “nuclear structure” (10 unigenes).

GO enrichment analysis was used to assemble unigenes and provided defined ontologies to express gene product properties. According to the results of Nr annotation, the GO classification of unigenes was generated using the Blast2GO program. We categorized 24,368 unigenes by using GO classification, yielding 60 functional groups across three main categories: biological process, cellular component, and molecular function (Figure 2B; Table S5). Among the 25 functional groups of the biological process category, “cellular process” (13,799 unigenes) and “single-organism process” (11,154 unigenes) had the largest proportions. Similarly, among the 18 functional groups of the cellular component category, “cell” (8865 unigenes) and “cell part” (8757 unigenes) were the most highly represented. In particular, “growth” was represented by 299 unigenes. Furthermore, among the 17 functional groups of the molecular function category, “binding” (12,232 unigenes) and “catalytic activity” (8707 unigenes) were the most abundant.

The assembled unigenes of the potato grouper transcriptome were compared with the KEGG database by using BLASTx to identify the corresponding pathways. We consequently assigned 51,262 unigenes to 42 KEGG pathways (Figure 2C; Table S6); among the largest cluster was “Signal transduction” (10,979 unigenes), followed by “Cancer: Overview” (6682 unigenes), “Immune system” (5872 unigenes), “Global and overview maps” (5418 unigenes), and “Infection disease: Bacterial” (5196 unigenes). The three smallest clusters were “Membrane transport” (210 unigenes), “Metabolism of terpenoids and polyketides” (83 unigenes), and “Biosynthesis of other secondary metabolites” (26 unigenes).

A four-way Venn diagram plot (Figure 2D) presents the assembled unigenes annotated against the Nr, COG, KEGG, and SwissProt databases. The figure indicates that 22,031 unigenes were concurrently annotated on all four databases and that 14 were annotated in both KEGG and COG database, 2565 in Nr and KEGG databases, 58 in Nr and COG databases, 4371 in Nr and SwissProt databases, 1389 in KEGG and SwissProt databases, and 6 in COG and SwissProt databases.

#### 3.1.2. Functional SSR and SNPs Discovery

**Figure 3:**
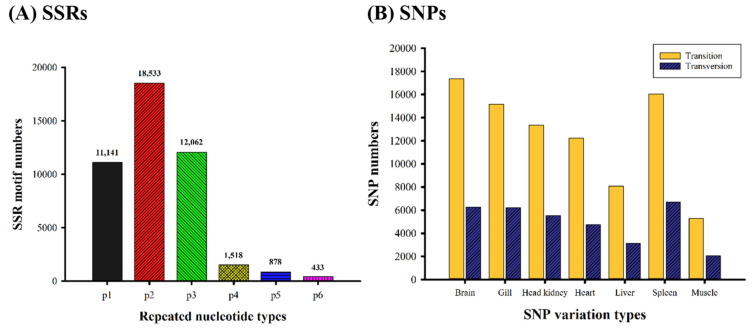
Distribution of different SSR motif types (**A**). The numbers of SNP variations in different tissues (**B**). Abbreviations: p1, mononucleotide; p2, dinucleotide; p3, trinucleotide; p4, tetranucleotide; p5, pentanucleotide; p6, hexanucleotide.

A total of 44,565 SSR (Figure 3A) were identified from the potato grouper transcriptome by using the MISA software, including mono-, di-, tri-, tetra-, penta-, and hexanucleotide repeats. The most abundant repeat motif was dinucleotide (n = 18,533; most commonly AC/GT, followed by AG/CT and AT/AT), followed by trinucleotide (n = 12,062; most commonly AGG/CCT, followed by AGC/CTG and ATC/ATG), mononucleotide (n = 11,141), tetranucleotide (n = 1518), pentanucleotide (n = 878), and hexanucleotide (n = 433).

We identified 122,220 SNPs (Figure 3B) (87,542 transitions and 34,678 transversions) from mapping sequencing reads to assembled unigenes by using HISAT software. Under seven cDNA libraries, the total numbers of the two transition types A/G and C/T were 45,080 and 42,462, respectively, and the total numbers of the transversion types A/C, A/T, G/C, and G/T were 8824, 8144, 8809, and 8901, respectively. The transition/transversion (Ts/Tv) ratio was approximately 2.52.

### 3.2. Growth Experiment

The initial and final BW, BL, and TL for 180 juveniles of potato grouper after the 90-day growing period are presented in Table S8. The average initial BW, BL, and TL across the six groups were 3.51 ± 0.71 g, 5.07 ± 0.38 cm, and 5.91 ± 0.43 cm, respectively and the average final BW, BL, and TL were 79.72 ± 12.91 g, 13.81 ± 0.72 cm, and 16.66 ± 0.86 cm, respectively. The differences between initial and final values were significant. The survival rate was 97.78%.

### 3.3. Molecular Markers of the Potato Grouper

#### 3.3.1. Genetic Diversity of Functional and Nonfunctional SSR

We selected 67 functional and 14 nonfunctional SSR markers (Table S7) from the potato grouper transcriptome and the reference related to grouper species (*E. fuscoguttatus* and *Plectropomus leopardus*), among which two growth-related molecular SSR were from our previous experiment. These markers were analyzed in terms of genetic diversity, broodstock management, and growth-related traits. PCR annealing test and capillary electrophoresis revealed 81 SSR, 54 of which were credible for use in the following analysis. Finally, 13 of 54 credible SSR in juveniles demonstrated polymorphism, including 10 functional SSR (Unigene18343, Unigene24547, Unigene26767, Unigene43252, Unigene64240, CL2428.Contig2, CL3784.Contig1, CL4125.Contig1, CL6953.Contig2, and mef2d_B) and three nonfunctional SSR (Efu_2–32, Efu_2–33, and Efu_6–1). We summarized 10 functional SSR markers with polymorphisms that were used to analyze the genetic diversity of juveniles (Table S9). The fragments of alleles of the 10 markers (Unigene18343, Unigene24547, Unigene26767, Unigene43252, Unigene64240, CL2428.Contig2, CL3784.Contig1, CL4125.Contig1, CL6953.Contig2, and mef2d_B) had lengths of 134/136 bp, 138/150/156/160 bp, 150/156 bp, 226/236 bp, 168/170/174 bp, 326/344 bp, 173/176 bp, 158/162/164 bp, 196/220 bp, and 407/409 bp, respectively. The number of genotypes was three, four, two, two, three, two, two, four, two, and three, respectively. In particular, Unigene24547 had 100% of the observed heterozygosity in juveniles. In addition, three nonfunctional SSR (Efu_2–32, Efu_2–33, and Efu_6–1) yielded two (156 and 160 bp), two (147 and 149 bp), and three (199, 203, and 209 bp) alleles, respectively, and the numbers of genotypes was two for all (156/156, 156/160 bp; 147/147, 147/149 bp; and 199/209, 203/209 bp, respectively).

The seven most abundant SSR polymorphisms (Unigene18343, Unigene24547, Unigene64240, CL4125.Contig1, Efu_2–32, Efu_2–33, and Efu_6–1) were analyzed in both broodstock and juveniles (Table 1; Table S10). In the broodstock, the alleles of seven markers (Unigene18343, Unigene24547, Unigene64240, CL4125.Contig1, Efu_2–32, Efu_2–33, and Efu_6–1) yielded 134/136 bp, 138–160 bp, 168–176 bp, 158–170 bp, 154–160 bp, 145–149 bp, and 99–215 bp, respectively. The number of genotypes was three, fourteen, nine, seventeen, nine, four, and nineteen, respectively. Across the four markers, the average N_A_ and N_G_ were 5.3 ± 2.8 and 10.7 ± 6.2, respectively, average *H_O_* and H*_E_* was 0.7 ± 0.2 and 0.6 ± 0.2, respectively, and the average PIC and *F_IS_* were 0.6 ± 0.2 and −0.1 ± 0.1, respectively.

In the juveniles (Table 1), across the seven SSR markers, the average N_A_ and N_G_ were 2.7 ± 0.8 and 2.9 ± 0.9, respectively, and the average *H_O_* and PIC was 0.7 ± 0.2 and 0.5 ± 0.1, respectively. The genetic diversity analysis indicated that broodstock and juveniles cosymbolized a high polymorphism, and the broodstock was at a crossbreeding stage. The genotypes of the seven SSR markers across the broodstock and juveniles demonstrated that juveniles were a full-sib family (Figure 1; Figure S3).

#### 3.3.2. SNPs

In total, 46 SNP markers were selected from the transcriptome, which was analyzed with Agena MassARRAY. The sequencing results revealed that all SNP assays had a 90% call rate, and the frequency of poly-and monomorphic SNP genotyping was derived from the 46 SNPs according to the nucleobase proportion of molecular weight (Figures S4A,B and S5A,B). Moreover, 38 of 46 SNPs were successfully genotyped; however, PCR amplification, clustering, and genotyping failed in the remaining eight (Unigene3858, Unigene7476, Unigene9685, Unigene25283, CL1123.Contig1, CL1123.Contig5, CL3051.Contig2, and CL4543.Contig1) (Table S11). In the broodstock and juveniles, we clustered the 38 SNP arrays into three groups depending on whether they had three, two, or one genotype. We found that seventeen and two SNP assays in the broodstock and juvenile groups, respectively, could be clustered into three genotypes; five and nine SNP assays, respectively, into two genotypes; and 16 and 27 SNP assays, respectively, into one genotype. Furthermore, 16 and 27 SNP assays, respectively, were homozygous and 22 and 12 SNP assays, respectively, were heterozygous. Among them, Unigene15043 in the juvenile group had only one heterozygous genotype (TA).

According to the genotype assays, AutoCluster was used to cluster homozygotes and heterozygotes into two groups to draw a two-dimensional graph. Of the 46 SNP assays in the broodstock and juveniles, 22 and 11 SNP assays, respectively, exhibited polymorphism; of them, 11 were the same between the groups (Unigene7626, Unigene15453, Unigene18387, Unigene18772, Unigene22300, CL2428.Contig3, CL2880.Contig2, CL4922.Contig6, CL5621.Contig2, CL7963.Contig2, and CL8791.Contig3) (Table 2; Figure S6A,B).

#### 3.3.3. Correlation Between Genotypes and Traits

**Figure 4:**
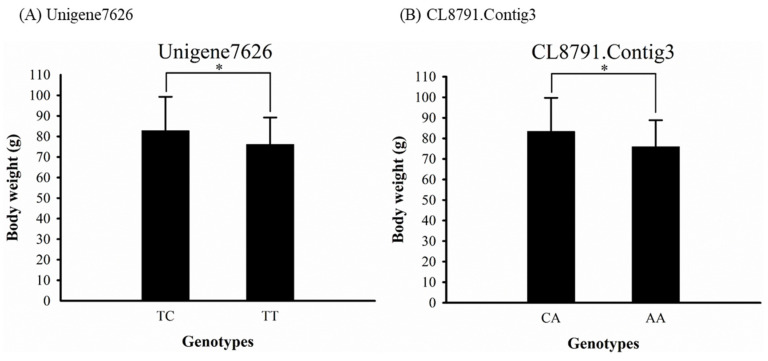
Number of juveniles with different genotypes at the two SNPs, Unigene7626 (**A**) and CL8791.Contig3 (**B**). The genotypes of the two SNPs were significantly different in terms of body weight based on one-way ANOVA (* *p* < 0.05).

In juveniles, BW was highly correlated with BL (R ^2^ = 0.8821) and TL (R^2^ = 0.8843) (Figure S7A,B). Thus, we analyzed the correlation of the 11 SNPs with BW (Table 2). Two SNP markers, Unigene7626 (*p* = 0.046) and CL8791.Contig3 (*p* = 0.032), were significantly correlated with growth traits (*p <* 0.05) (Figure 4A,B). For Unigene7626, the TC genotype had a significantly larger body size (*p* = 0.017) than the TT genotype. For CL8791.Contig3, the AA genotype had a significantly smaller body size (*p* = 0.014) than the CA genotype. The alignment of the SNP sequences with Nt annotation implied that two SNPs were located in 3′-UTR and nonsynonymous coding regions within the alpha cardiac muscle actin (*ACTC1*) and pericentrin (*PCNT*) genes, respectively.

### 3.4. Gene Expression in Different Tissues

For six juveniles from the FG and SG (control) groups, gene expression was examined for the brain, liver, and muscle tissues. Beta-actin was used as a reference gene for normalizing real-time PCR data in the groups for Unigne7626 (*ACTC1*) and CL8791.Contig3 (*PCNT*), the two genes tested, and their expression levels were compared between the two groups (Figure S8). Compared with the SG (control) group, Unigene7626 (*ACTC1*) was downregulated in all the three tissues of the FG group; moreover, CL8791.Contig3 (*PCNT*) was upregulated in the brain and liver tissues and downregulated in the muscle tissue of the FG group.

## 4. Discussion

### 4.1. The Potato Grouper Transcriptome and Candidate Functional Molecular Markers

The diversity of grouper species yields the advantages of interbreeding and dominant inheritance, such as the *E. fuscoguttatus* × *E. lanceolatus* hybrid grouper [51]. The potato grouper is a novel cultured grouper species in Taiwan with a potentially high growth performance; however, it is occasionally polycultured with different grouper species, possibly causing unexpected hybrids. This increases the degree of complexity in broodstock management. In such a case, molecular markers are helpful for tracking and managing the broodstock [52]. In the present study, we discovered that the available molecular markers form a close genetic relationship, meaning that they had not only low genetic diversity and commonality but also a smaller number of markers. Next-generation sequencing is efficient and productive for the development of numerous molecular markers in nonmodel animals. Whole-genome sequencing (WGS) generates a complete gene database of the species. However, establishing a *de novo* transcriptome database of nonmodel aquaculture species is more cost-effective and faster than WGS [53]. In addition, the applications of microarray, MassArray, RNA-seq, ddRAD-seq, and SLAF-seq have been adequately researched in aquaculture animals [54,55,56]. Furthermore, *de novo* transcriptome yields numerous EST molecular markers (SSR and SNPs) because of the annotation of different functional databases (COG, GO, KEGG, and SwissProt) [57], which may facilitate understanding of the complex trait mechanisms, genetic management, and genetic improvement of stocks [58].

In aquaculture species, the construction of a cDNA library at the transcriptome level can be commonly achieved with numerous methods, including pooling the same tissues from different individuals in the same treatment, using one tissue from an individual, and pooling all tissues as one sample [59,60,61,62]. The choice of the approach depends on factors such as cost, the purpose of research, rare species or individuals, and minimization of interindividual variation. In the present study, because of the low population of potato groupers in the broodstock and lack of genetic bioinformation, a *de novo* transcriptome was created from an individual fish using seven cDNA libraries to discover expressed sequences, which can be annotated with functional genes and show tissue-specific diversity.

A transcriptome is a powerful tool that provides not only different functional annotations of a bioinformatic expression gene between distinct samples but also molecular markers for application in aquaculture, conservation, fisheries management, genetics, and breeding, as well as correlation with target traits [63,64,65]. In the present study, RNA-seq was used to analyze the phenotypes of growth performance because ESTs may directly influence organism performance and also built the cornerstone of the database for other economical traits [66,67,68,69,70,71]. For example, considerable research using RNA-seq has been performed on the economically important aquaculture species of Atlantic salmon, rainbow trout, turbot, gilthead seabream, and Pacific white shrimp, which helps comprehension of the variation of transcripts between cells, tissues, ontogenetic, food nutrition, and environmental conditions and facilitates analysis if those traits or performance indicators are correlated with the polymorphic markers [72,73,74,75,76,77,78,79]. The common economic aquaculture grouper species—orange-spotted grouper, giant grouper, and hybrid grouper (*E. fuscoguttatus* × *E. lanceolatus*)—have been evaluated for growth trait performance, virus resistance, dietary supplementation, and temperature challenge, and the results have revealed some underlying molecular mechanisms [80,81,82,83]. Compared with our database, finding out growth-related functional annotation and molecular mechanisms are both shared in the GH/IGF axis and its downstream signaling pathways. However, these studies have focused on gene function and its integrated signal pathways; by contrast, transcripts identified using a transcriptome profile may influence target traits through various genes [84,85,86].

The potato grouper database was helpful in understanding its functional mechanisms/pathways, gene regulation in different tissues, and correlation of genotypes with traits. We screened the EST molecular markers derived from the transcriptome, which can be applied to genetic management and genetic diversity and growth-related analyses of potato grouper. These markers can be surveyed and used to verify performance by not only planning breeding schemes arranged in genotypes of EST markers to the next generation but also using CRISPR-Cas9 to transfect the target genes artificially to enhance transgenic fish behavior [87,88,89,90,91]. In addition, RNA-seq rapidly yielded numerous EST molecular markers that can be used in future studies on not only growth performance but also other traits, such as disease resistance, stress, and environmental change [92,93,94,95,96,97]. These useful functional molecular markers can provide a more efficient and systematic genetic method for artificial selection in hatchery [98].

### 4.2. Genetic Management, Genetic Diversity, and Growth-Related Molecular Markers in the Potato Grouper

Because of the lack of genetic information, RNA-seq was suitable to generate large amounts of molecular markers for nonmodel aquaculture species [99,100,101]. These annotations of functional genes and markers were highly conserved and potentially used in other similar species [102]. In Taiwan, especially in the early stages of breeding, inbreeding and interbreeding are common in hatcheries, without complete records; however, this problem can be resolved and managed through molecular biotechnology. SSR is widely used in genetic management, parentage analysis, genetic diversity, marker-assisted breeding selection [103], for differentiating population structures between released and wild species [104,105], conservation [106], and fisheries management [107]. Genetic diversity was high in every generation and breed in which we used molecular markers to trace, manage, and prevent inbreeding and intra- and interspecies interbreeding [108,109,110]. We discovered that functional (type I) and nonfunctional (type II) SSR markers have low genetic diversity in breeding selection, with functional SSR markers derived from candidate stocks and juveniles being more sensitive than nonfunctional SSR markers [111]. However, an organism cannot select the best genotype–phenotype combination, probably because various genotypes may be lost during artificial selection with functional molecular markers [112,113]. Hence, using functional SSR markers with polymorphism is critical because they directly influence gene function and because they may be easily missed during the breeding process. In addition, genomic selection can help to not only track signatures of artificial selection and genetic diversity of stocks in the domestication of farmed species [114] but also perform parentage analysis, enabling breeders to trace back to the broodstock with genotypes of specific molecular markers [115,116,117]. The 21 functional molecular markers across broodstock and juveniles identified in the present study demonstrated that the number of alleles and genotypes in the juveniles sharply declined after mating. Selection breeding for economical traits is the first goal of a breeder; hence, maintaining the high variability of genes and population of genetic variation becomes important. The functional SSRs (mentioned above) are directly related to genes that their highly variable produces individual differences which are influentially bred with individual or population selection of potato grouper in the future.

Studies on Atlantic salmon (*Salmo salar*), orange-spotted grouper (*E. coioides*), and turbot (*Scophthalmus maximus*) have indicated that SNP markers are more related to growth traits than SSR are [118,119,120,121,122,123]. Therefore, growth-related SNP markers were selected from our transcriptome and referenced to other studies of the growth performance of fish species. Among them, Unigne7626 (*ACTC1*) and CL8791.Contig3 (*PCNT*) was evidently correlated with the growth traits of potato grouper. *ACTC1* plays a role in the cytoskeletal structure, cell motility, cell division, and intracellular movements and contractile processes in thin filaments [124]. In human studies, *ACTC1* mutations have been noted in heart diseases, including dilated cardiomyopathy and hypertrophic cardiomyopathy [125,126]. Lee et al. observed that *ACTC1* is upregulated in mud loaches during the growth stage [127], and Avey et al. suggested that *ACTC* expression in *D. rerio* alters the cardiac function and reduces aerobic efficiency [128]. Further research is warranted to determine whether *ACTC* mutation and expression during growth influence the growth performance of potato grouper. *PCNT* encodes the centrosome protein pericentrin, which contributes to the mitotic spindle for chromosomal segregation during cell division, thus influencing cell cycle progression and resulting in disorganized mitotic spindles and missegregation of chromosomes [129,130]. In Atlantic salmon, the SNP AX88270804 within *PCNT* may be associated with several growth traits, especially fat percentage, which has an approximate genetic variation of 4%, whereas the AA/GA/GG genotype of *PCNT,* which is associated with faster growth, is also associated with increased fatness [131]. In the current study, *PCNT* genotypes were also correlated with body weight: phenotypically, the CA genotype had the greatest growth, whereas the AA genotype had the smallest. However, these two SNP markers were not differentially expressed in different tissues between FG and SG groups, which phenotype may perform with polygenic inheritance [132] or epigenetics [133].

## 5. Conclusions

Our transcriptome analysis of a novel grouper species, potato grouper, provided useful bioinformation. The numerous molecular markers will facilitate genetic management, parentage analysis, genetic diversity in stocks, and complex and polygenic traits research; our findings thus established a basis for future genetic studies on potato groupers. The SNPs were within the *ACTC* and *PCNT* genes, which have potentially relevant functional connections to growth traits. Further breeding schemes of these candidate growth-related genes are warranted to identify putative causative variation.

## Figures and Tables

**Table 1:** Genetic diversity of seven SSR in broodstock and juveniles, and correlation with growth trait.

Broodstock								
Locus ^a^	N	N_A_	N_G_	*H_O_*	*H_E_*	PIC	*F_IS_*	HWE
Unigene18343	80	2	3	0.463	0.393	0.396	−0.177	ns
Unigene24547	80	7	14	0.800	0.710	0.715	−0.126	ns
Unigene64240	80	4	9	0.788	0.694	0.698	−0.135	ns
CL4125.Contig1	80	7	17	0.825	0.749	0.753	−0.102	ns
Efu_2–32	80	4	9	0.575	0.524	0.528	−0.097	**
Efu_2–33	80	3	4	0.513	0.502	0.505	−0.021	ns
Efu_6–1	80	10	19	0.738	0.831	0.836	0.112	**
Mean	80.0	5.3	10.7	0.7	0.6	0.6	−0.1	
±SD	±0	±2.8	±6.2	±0.2	±0.2	±0.2	±0.1	
**Juveniles**								
**Locus ^a^**	**N**	**N_A_**	**N_G_**	***H_O_***		**PIC**		
Unigene18343	94	2	3	0.511		0.501		
Unigene24547	94	4	4	1.000		0.753		
Unigene64240	94	3	3	0.574		0.423		
CL4125.Contig1	94	3	4	0.713		0.631		
Efu_2–32	94	2	2	0.479		0.366		
Efu_2–33	94	2	2	0.521		0.387		
Efu_6–1	94	3	2	1.000		0.627		
Mean	94	2.7	2.9	0.7		0.5		
±SD	±0.0	±0.8	±0.9	±0.2		±0.1		

^a^ Locus symbol is abbreviated according to the Unigene ID from the transcriptome database. Abbreviations: N, number of individuals analyzed; N_A_, number of alleles observed; N_G_, number of observed genotypes; *H_O_*, observed heterozygosity; *H_E_*, expected heterozygosity; PIC, polymorphic information content; *F_IS_*, individual fixation index; HWE, Hardy–Weinberg equilibrium (** *p* < 0.01 for the locus being out of the HWE); ns, not significant for HWE.

**Table 2:** Genotypes of 11 SNPs and their correlation with growth traits.

Assay ^a^	SNP	Amino Acid	Genotypes of Juveniles	Correlation with Growth Traits	Genotypes of Parent	
					Female	Male
Unigene7626	T/C	I/T	TT/TC	0.046 *	TT	TC
Unigene15453	A/G	H/R	GG/AA	0.253	GA	GA
Unigene18387	C/A	H/N	AA/CA	0.447	CA	AA
Unigene18772	C/T	P/L	CC/CA	0.475	CA	CC
Unigene22300	G/T	STOP/L	GG/GT/TT	0.155	GT	GT
CL2428.Contig3	C/G	T/R	CC/CG	0.259	CG	CC
CL2880.Contig2	C/T	S/L	CC/CT/TT	0.746	CT	CT
CL4922.Contig6	A/G	S/G	AA/AG	0.580	AA	GA
CL5621.Contig2	G/A	R/K	AA/GA	0.318	AG	GG
CL7963.Contig2	T/G	V/G	GT/TT	0.290	TT	GT
CL8791.Contig3	A/C	K/Q	AA/CA	0.032 *	AA	CA

^a^ Locus symbol is abbreviated according to the Unigene ID from the transcriptome database. The genotypes of SNPs were calculated using one-way ANOVA (* *p* < 0.05) with the correlation of growth traits.

## Data Availability

The data presented in this study are available within the article. If needed, supplementary material is available on request from the corresponding author.

## Supplementary Materials

The following are available online at https://www.mdpi.com/2079-7737/10/1/36/s1, Figure S1: Statistical length of the all-Unigene distribution. Figure S2: Distribution of annotated species that is statistics with Nr annotation. Figure S3: Graph of seven times of growth measurement. Figure S4: Percentage of genotyping call rate% well 1 (A) well 2 (B). Figure S5: Frequency of polymorphic and monomorphic SNPs genotyping derived from 46 genes well 1 (A) well 2 (B). Figure S6: Genotypes of MassARRAY in the two SNPs, Unigene7626 (A) and CL8791.Contig3 (B). Figure S7: The correlation (R^2^) body weight with body length (A) and total length (B). Figure S8: Relative expression of Unigene 7626 and CL8791. Contig3 in the offspring brain, liver, and muscle tissues. Table S1: Statistics of clean reads after filtering. Table S2: Statistics of the sequencing data after quality trimming. Table S3: Results of all-Unigene annotated to different databases. Table S4: Statistics of unigenes in COG category. Table S5: Statistics of unigenes in GO category. Table S6: Statistics of unigenes in KEGG category. Table S7: Primer lists of functional and nonfunctional SSR, SNP, and qRT–PCR. Table S8: Growth performance. Table S9: Genotypes of 13 functional SSR in juveniles. Table S10: Paternity analysis of broodstock and juveniles. Table S11: Genotypes of 46 SNP arrays from MassARRAY.
