# Supplementary material for: Development of EST-Molecular Markers from RNA Sequencing for Genetic Management and Identification of Growth Traits in Potato Grouper (Epinephelus tukula)
Source: Biology (Basel). 2021 Jan 7;10(1):36. doi: 10.3390/biology10010036 (PMC7825770; doi:10.3390/biology10010036)
Supplement: Supplementary file 1 [file biology-10-00036-s001.zip › Potato grouper_Figure S1-S8.pdf]

Supplementary Materials

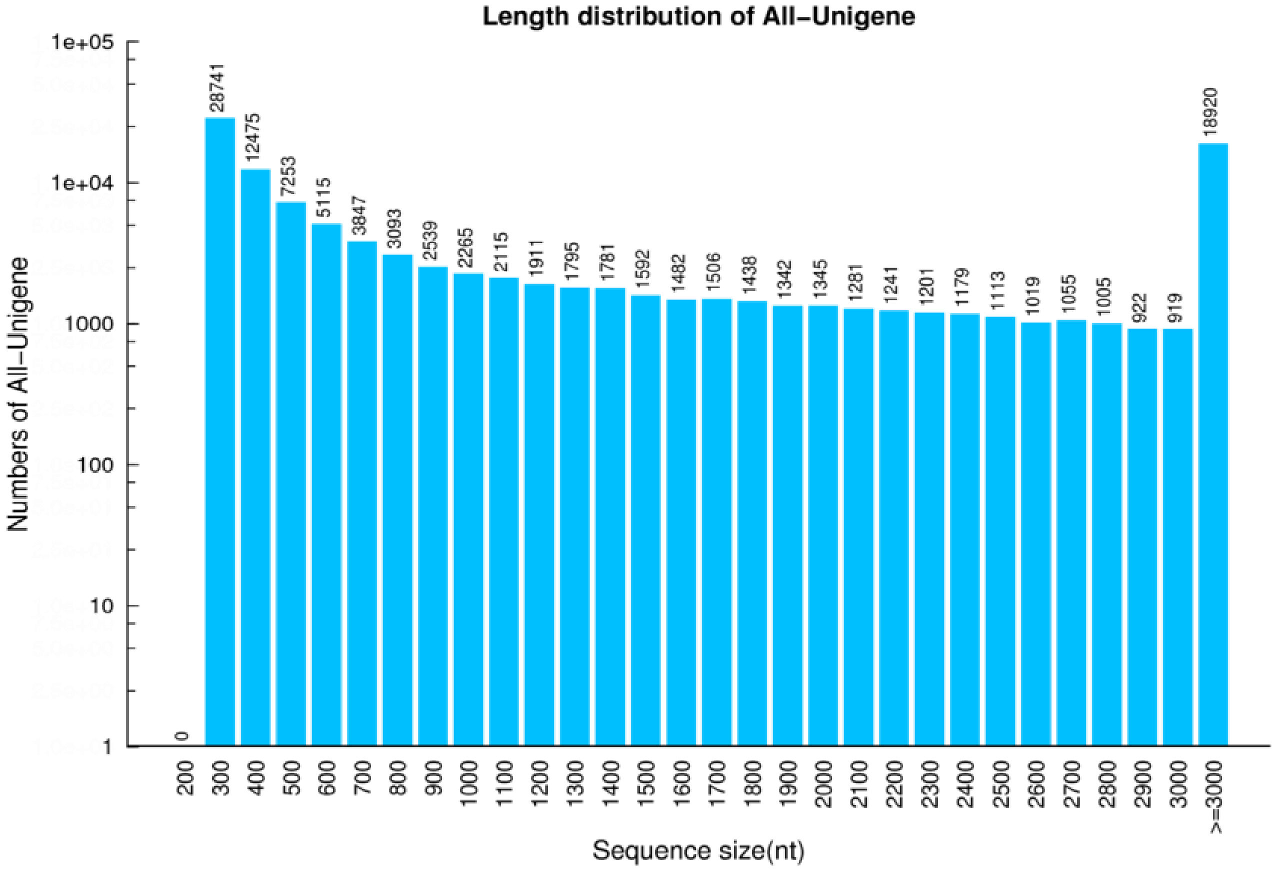

Figure S1. Statistical length of the all-unigene distribution. X-axis represents the length of transcripts. Y-axis represents the number of transcripts.

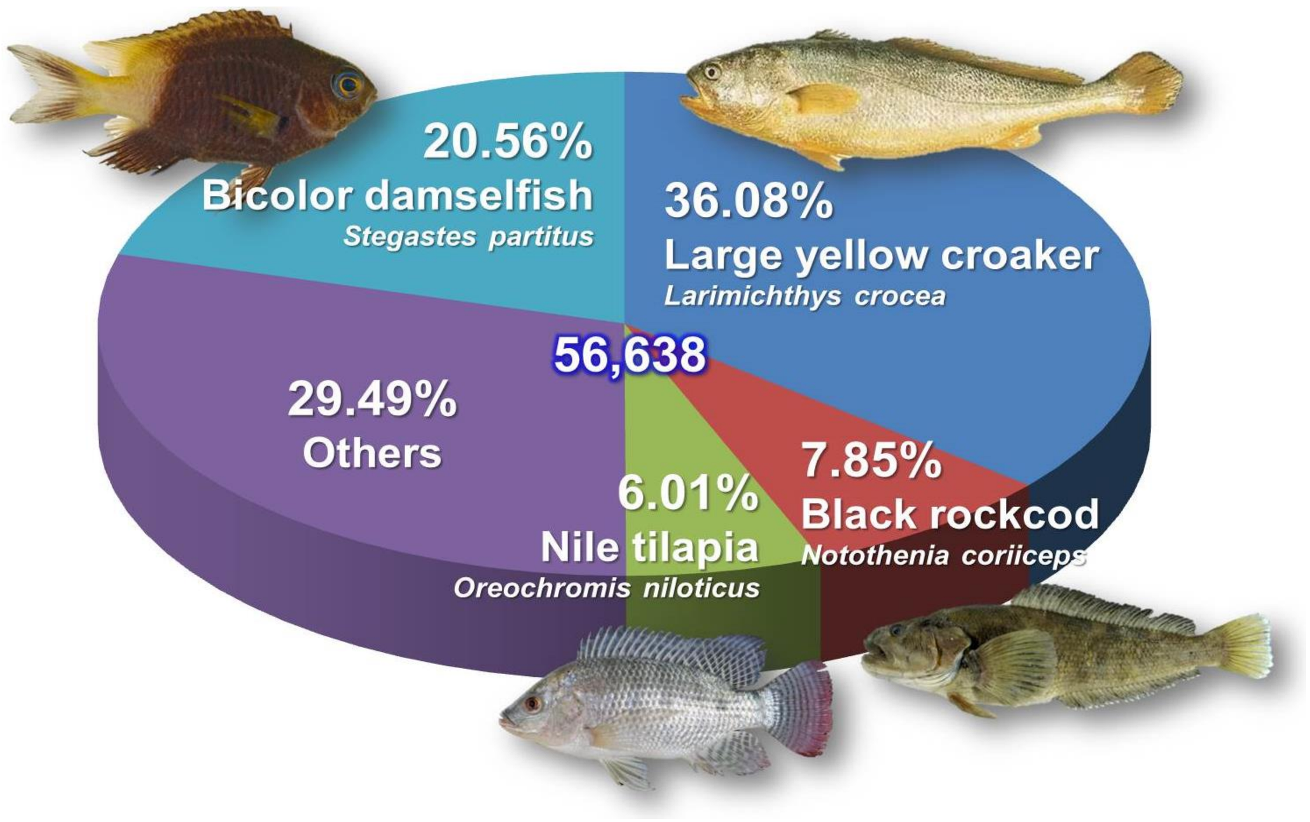

Figure S2. Distribution of annotated species that is statistics with Nr annotation. *Oreochromis niloticus* (6.01 %); *Larimichthys crocea* (36.08 %); *Notothenia coriiceps* (7.85 %); *Stegastes partitus* (20.56 %); other (29.49 %).

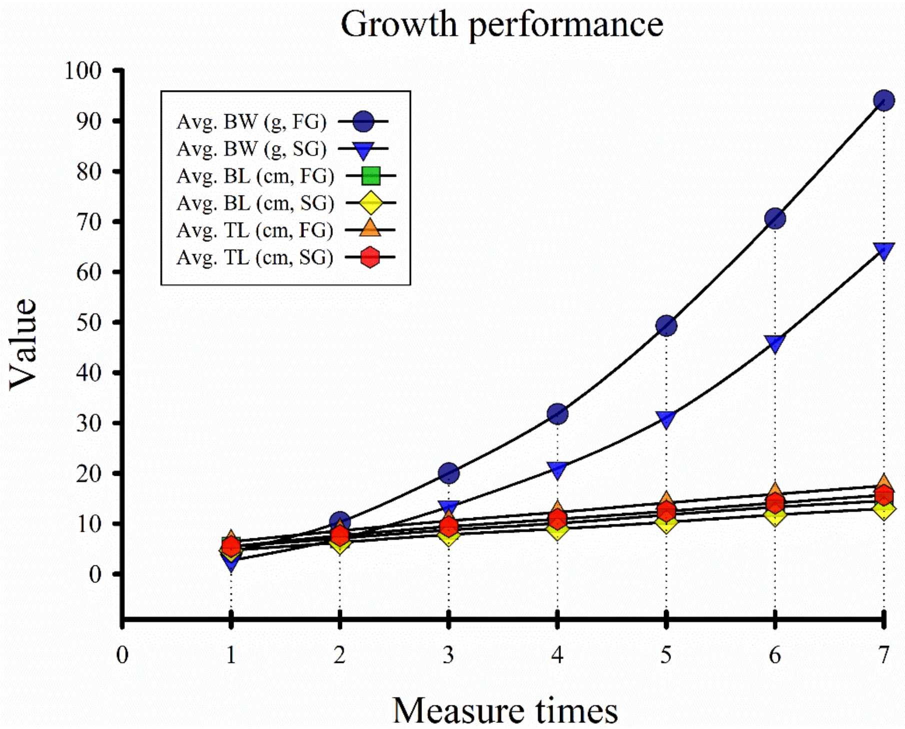

Figure S3. Graph of seven times growth measurement. Avg. BW: average of body weight in each measurement; Avg. BL: average of body length in each measurement; Avg. TL: average of total length in each measurement.

(A) Well 1

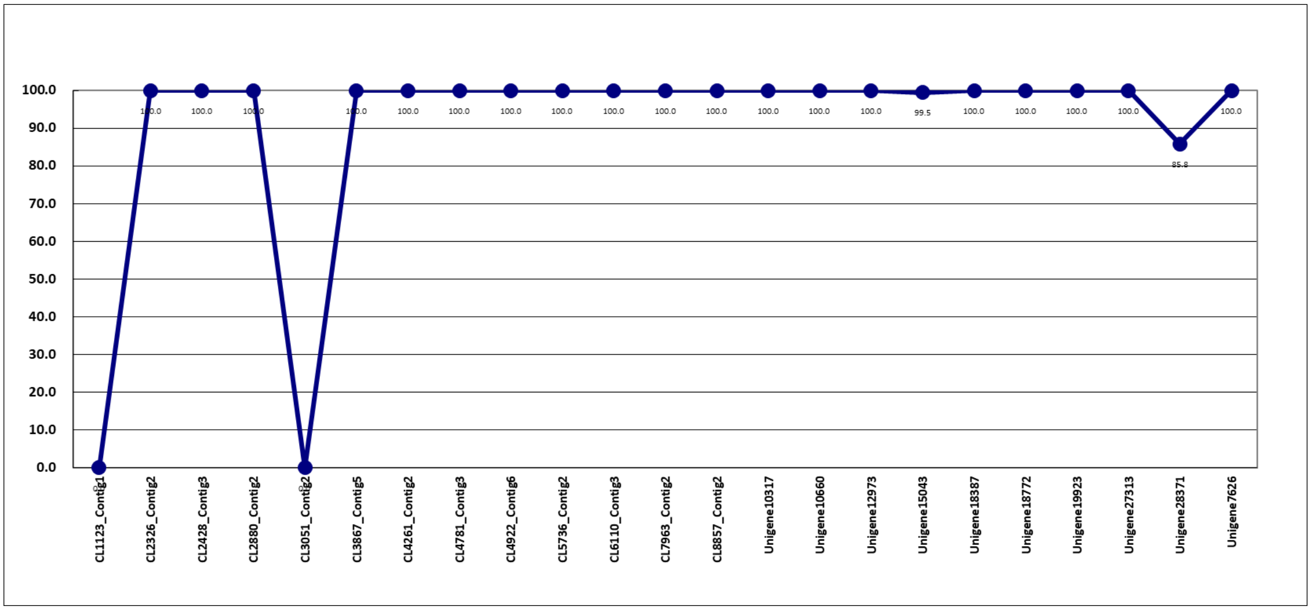

(B) Well 2

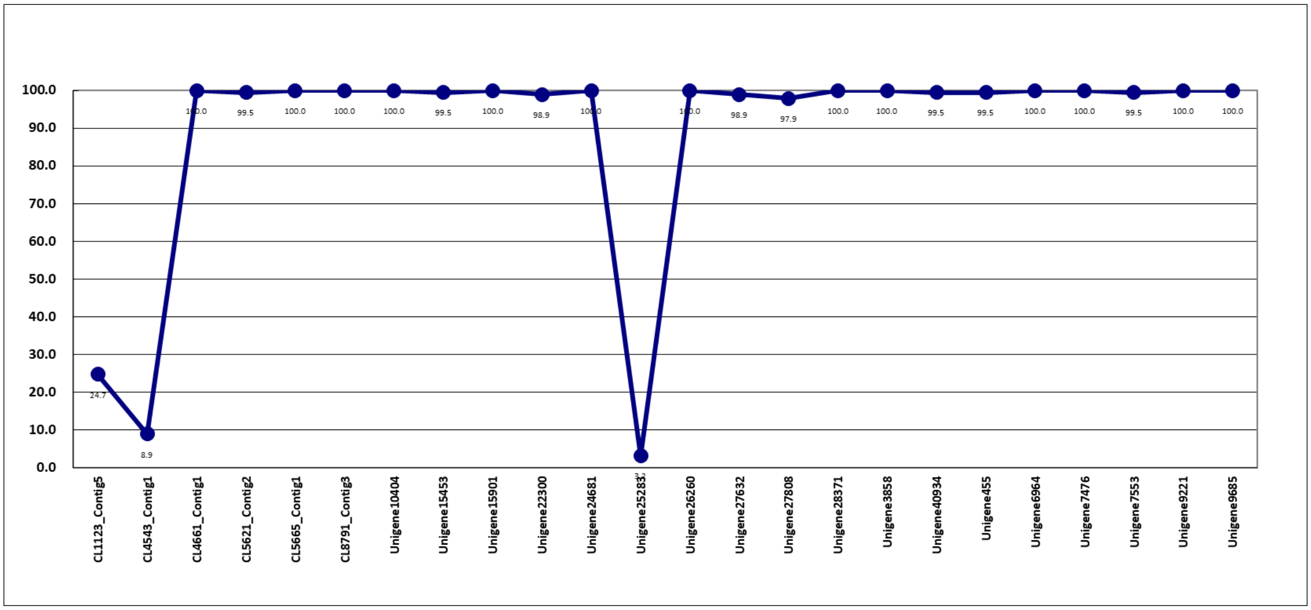

Figure S4. Percentage of genotyping call rate% Well 1 **(A)** Well 2 **(B)**.

(A) Well 1

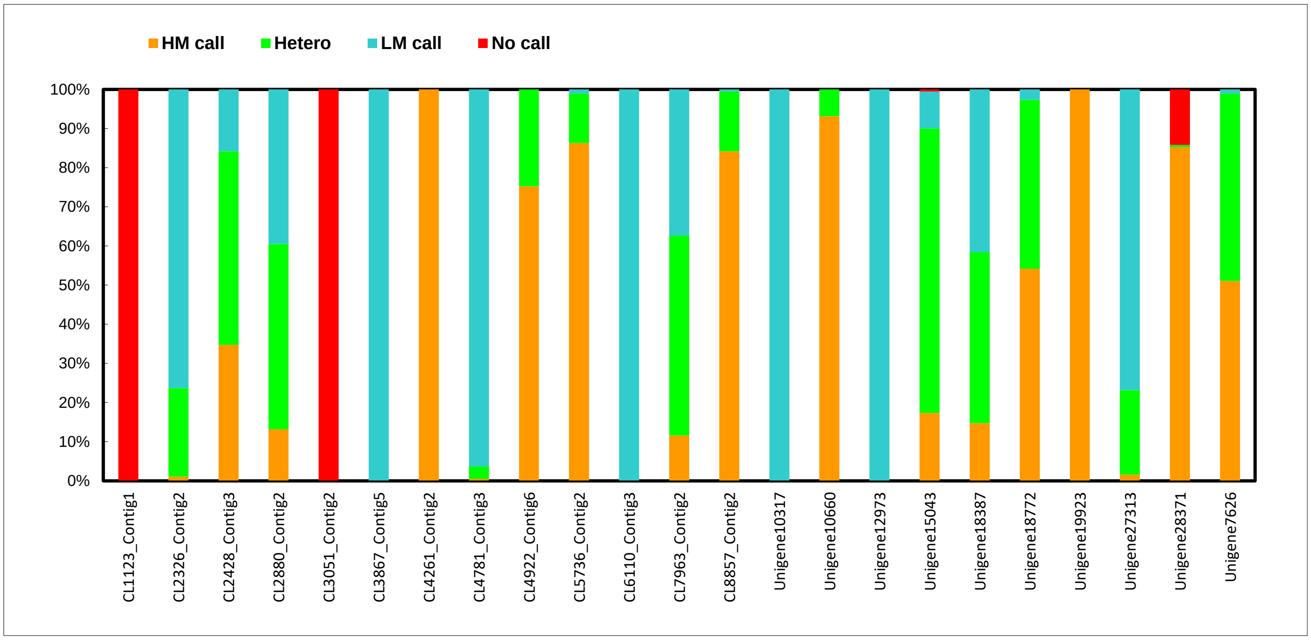

(B) Well 2

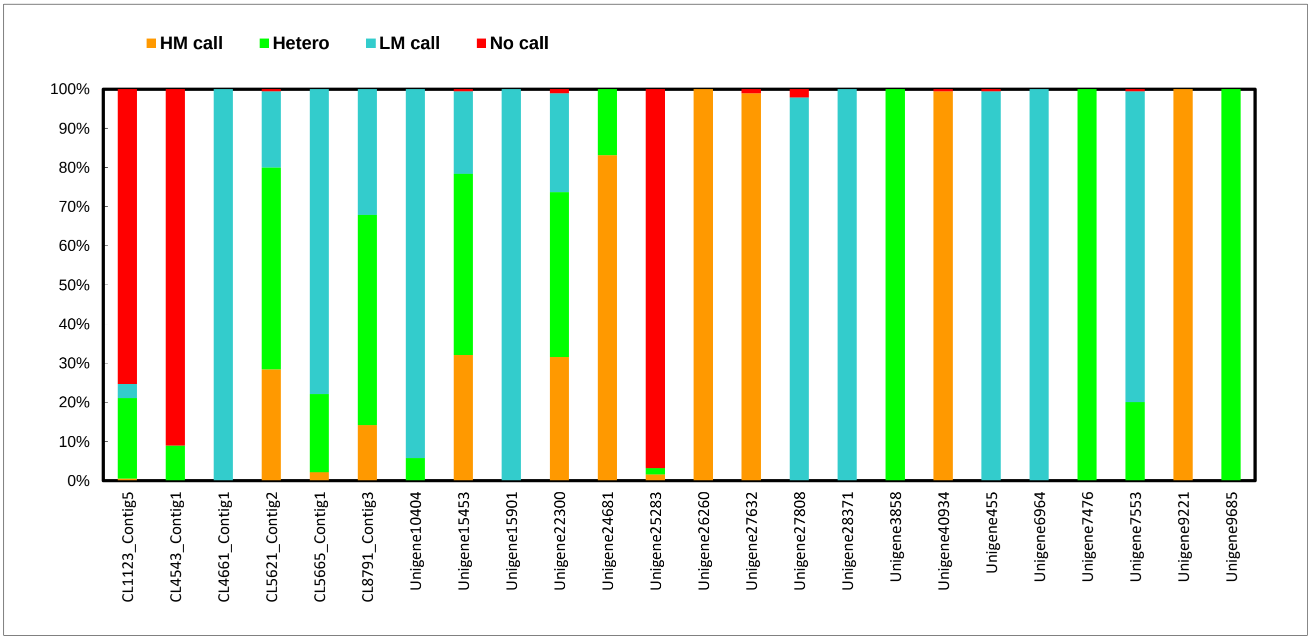

Figure S5. Frequency of polymorphic and monomorphic SNPs genotyping derived from 46 genes Well 1 **(A)** Well 2 **(B)**. HM call: High mass allele genotype calling; Hetero: Heterozygous genotype calling; LM call: Low mass allele genotype calling; No Calls: Failed for genotyping.

(A) Unigene7626

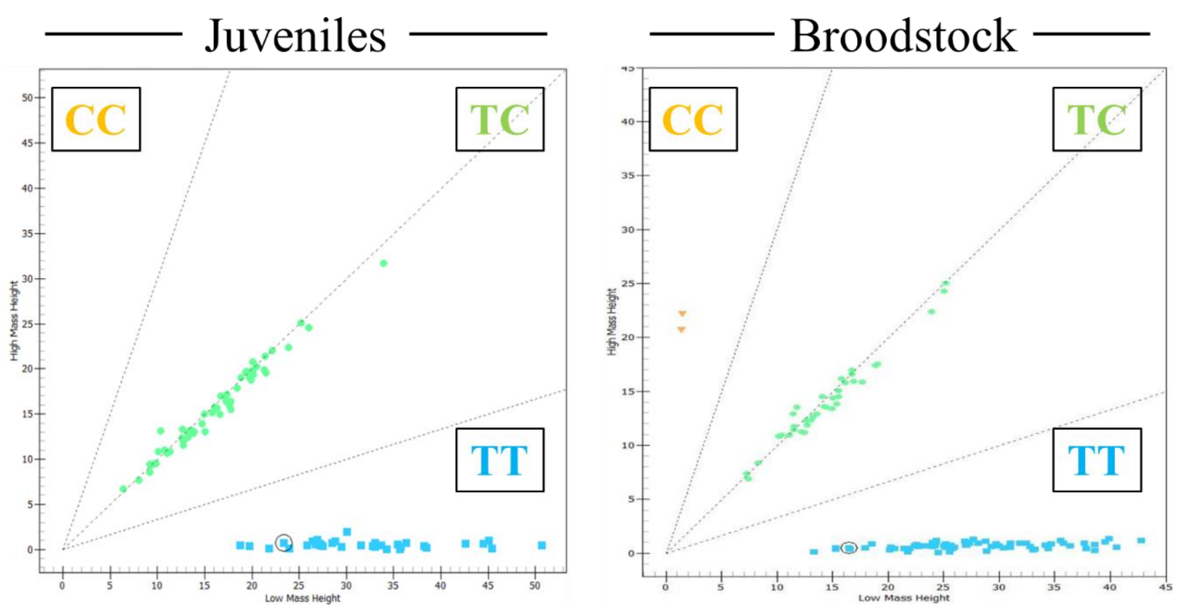

(B) CL8791.Contig3

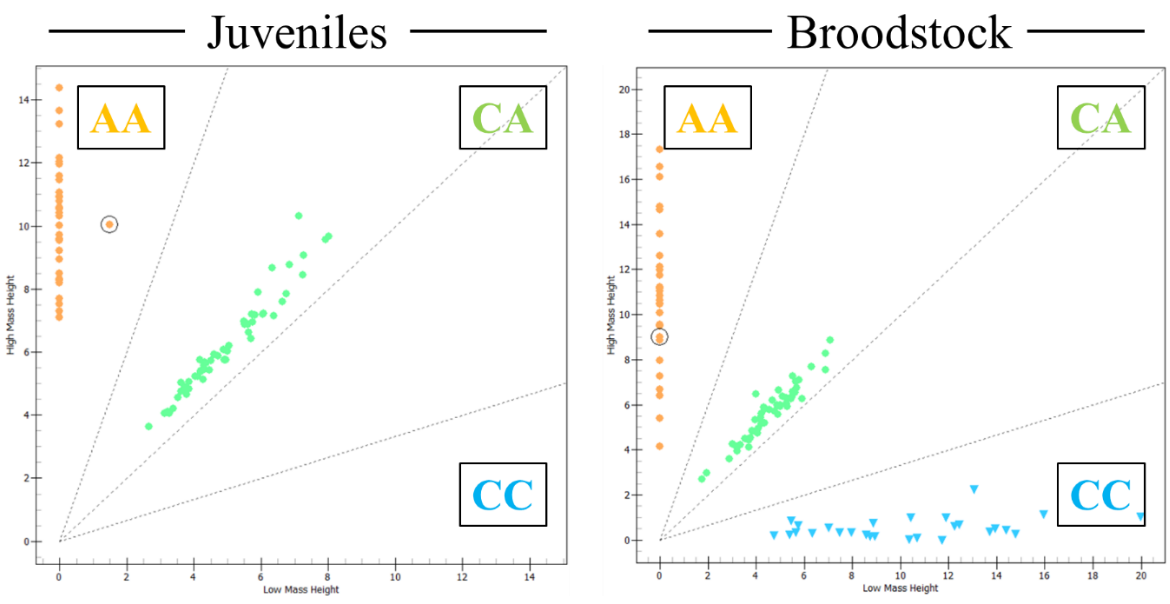

Figure S6. Genotypes of MassARRAY in the two SNPs, Unigene7626 **(A)** and CL8791.Contig3 **(B).**

(A) Body length / Body weight

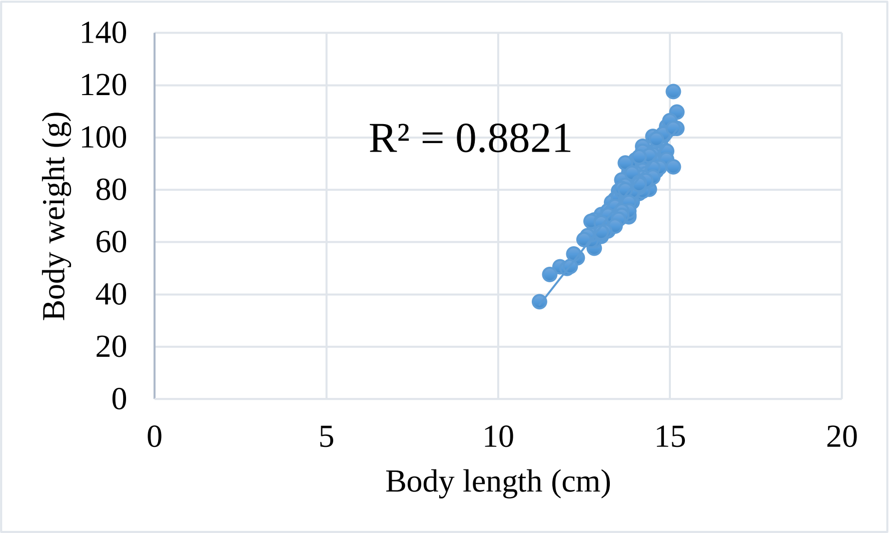

(B) Total length / Body weight

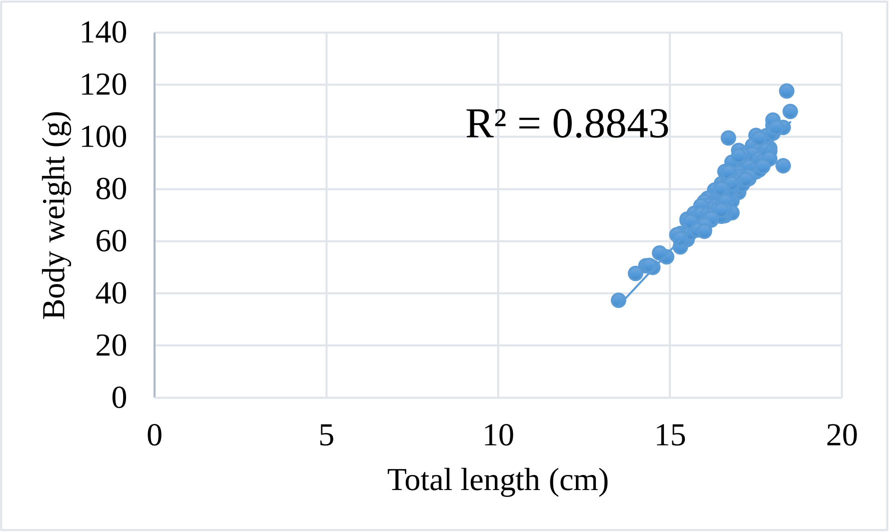

Figure S7. The correlation (R^2) body weight with body length **(A)** and total length **(B)**.

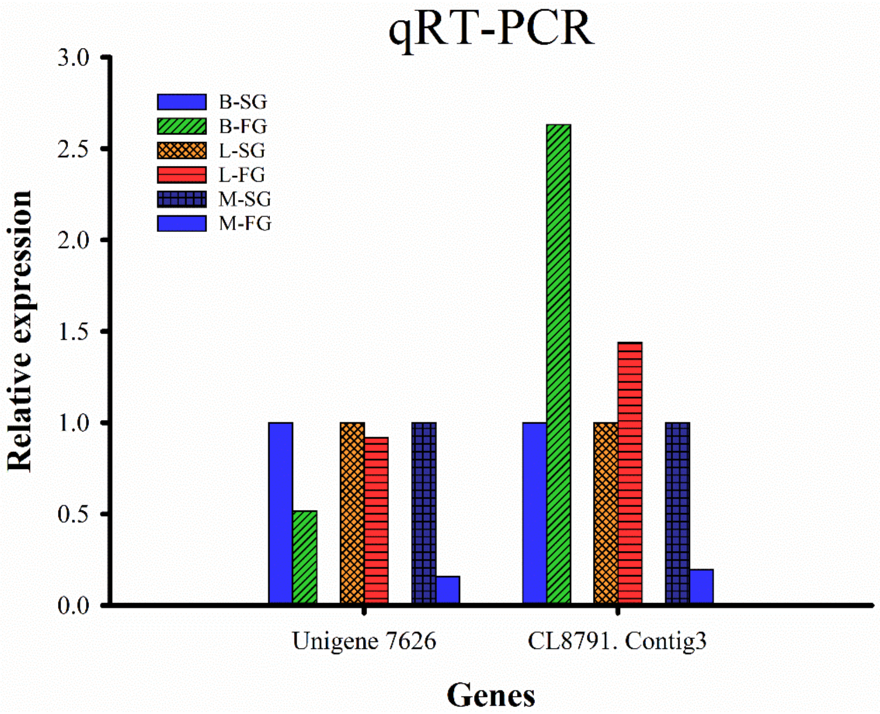

Figure S8. Relative expression of Unigene 7626 and CL8791. Contig3 in the offspring brain, liver, and muscle tissues. Cp values from 3 independent replicates in FG and SG group which separately were used to calculate the expression in two genes. The FG group normalized by the SG group and relative to it using the $\log_2$ (fold change). Abbreviation: B, Brain; L, Liver; M, Muscle; FG, Fast Growth; SG, Slow Growth.
